# Implementation of screening criteria for inflammatory bowel disease in patients with spondyloarthritis and its association with disease and endoscopic activity

**DOI:** 10.1007/s10067-022-06297-7

**Published:** 2022-09-02

**Authors:** Jaiber Gutiérrez-Sánchez, Viviana Parra-Izquierdo, Cristian Flórez-Sarmiento, Diego Alejandro Jaimes, Juliette De Ávila, Juan Manuel Bello-Gualtero, Alejandro Ramos-Casallas, Lorena Chila-Moreno, César Pacheco-Tena, Adriana Beltrán-Ostos, Philippe Chalem-Choueka, Wilson Bautista-Molano, Consuelo Romero-Sánchez

**Affiliations:** 1 Rheumatology and Immunology Department/Clinical Immunology Group, Hospital Militar Central, Transversal 3ª # 49-00, Bogotá, Colombia; 2 School of Medicine, Clinical Immunology Group, Universidad Militar Nueva Granada/Hospital Militar Central, Transversal 3ª # 49-00, Bogotá, Colombia; 3 School of Dentistry, Cellular and Molecular Immunology Group/INMUBO, Universidad El Bosque, Av. Carrera 9 # 131A-02, Bogotá, Colombia; 4 Gastroadvanced SAS IPS, Carrera 23 # 45C-31, Bogotá, Colombia; 5 Clínicos IPS, Carrera 15 # 98-29, Bogotá, Colombia; 6 Investigación Y Biomedicina De Chihuahua S.C., Calle 16 # 1600, Chihuahua, Chihuahua México; 7 Fundación Instituto de Reumatología Fernando Chalem, Calle 73 # 20A - 27, Bogotá, Colombia

**Keywords:** Calprotectin, Inflammatory bowel disease, Ileocolonoscopy, Spondyloarthritis

## Abstract

There is little literature on the implementation of screening criteria for inflammatory bowel disease (IBD) in patients with spondyloarthritis (SpA). This study aimed to apply IBD screening criteria in a group of patients with SpA without IBD diagnosis and correlate them to endoscopic findings and disease activity. A total of 82 patients with SpA were included. The IBD screening test and ileocolonoscopy with digital chromoendoscopy with magnification and histological analysis were performed. The data were analysed with Chi-square test/Fisher’s exact test and multiple correspondence analysis. The major screening criteria found in 48.7% of the patients were associated with a history of infection (*p* = 0.037). Rectal bleeding was associated with the diagnosis of ankylosing spondylitis, acute inflammation, enthesitis and tissue architecture alteration in the ileum (*p* < 0.050). Diarrhoea was associated with a higher disease activity score (*p* = 0.02). Minor screening criteria were associated with painful inflammatory joint (*p* = 0.05), high disease activity score (*p* = 0.001) and high calprotectin levels (*p* = 0.050). Abdominal pain (36.9%) was associated with axial/peripheral compromise (*p* = 0.017), inflammatory back pain (*p* = 0.01), enthesitis (*p* = 0.021), higher disease activity score (*p* = 0.023) and acute ileum inflammation (*p* = 0.046). Diarrhoea of 4 weeks and abdominal pain were the most prevalent major and minor screening criteria, respectively, being related to early manifestations of inflammatory bowel compromise and higher disease activity score. This screening test grants a chance of opportune referral of SpA patients from rheumatology to gastroenterology.

**Supplementary Information:**

The online version contains supplementary material available at 10.1007/s10067-022-06297-7

## Introduction

In Colombia, spondyloarthritis (SpA) has an estimated prevalence of 0.28% [1]. SpA is a group of chronic autoinflammatory diseases that can manifest with extra-articular symptoms [2]. Romero-Sánchez reported a high frequency of gastrointestinal symptoms in SpA [6]. Still, there is little literature describing gastrointestinal symptoms in patients with SpA without a diagnosis of inflammatory bowel disease (IBD) [3, 4].

Recognising this association between the intestine and joints impacts the timely diagnosis and the choice of therapy for patients [5]. Clinically evident IBD is observed in less 15% of ankylosing spondylitis (AS), and up to 60.0% of patients with SpA present subclinical inflammatory bowel involvement, which supports the importance of conducting endoscopic studies and the priority of evaluating associated gastrointestinal symptoms [6–8]. The application of IBD screening criteria in a group of patients with SpA without a diagnosis of IBD allows for detecting patients with subclinical IBD, becoming a valuable tool in prompt referral to gastroenterologists.

In our study, the objective was to describe the results of IBD screening instrument [9] implementation in Colombian patients with SpA and establish its association with early inflammatory findings of the intestinal mucosa.

## Materials and methods

### Study design and population

A cross-sectional study was carried out with non-probabilistic convenience sampling. Initially, 409 medical records were reviewed and preliminarily classified with SpA. Of those, 240 were selected meeting criteria according to the ESSG and ASAS group. Then, 180 Colombian patients were evaluated by rheumatologists, and finally, 82 were selected for application of the screening criteria proposed by Sanz et al. [9]. The screening criteria, endoscopic procedure, clinimetry, evaluation of faecal calprotectin, inflammatory markers, histological analyses and statistical analysis are described in Supplementary Annex 1.

## Results

This study included 82 patients, 54.9% men and 45.1% women (Table 1). According to the rheumatological variables, we found that 81.7% of the patients had radiographic axial SpA, 4.8% had reactive arthritis and 8.5% had psoriatic arthritis. Regarding the ASAS criteria, 81.7% met the criteria for axial involvement and 19.5% for peripheral involvement. On the other hand, high calprotectin levels were present in 24.4% of the patients, CRP was higher in 50%, and ESR was normal in 73.2% (Table 2).

**Table 1:** Demographic variables group of patients with spondyloarthritis

Variable	Patients (*n* = 82)	
	*n*	**%**
Sex		
Female	37	45.1%
Male	45	54.9%
Cigarette smoking		
Current smoker	5	6.1%
Former smoker	25	30.5%
Passive smoker	12	14.6%
Economy activity		
Student	5	6.1%
Employee	33	40.2%
Independent	20	24.4%
Retired	11	13.4%
Home	13	15.9%
Housing		
Own	42	51.2%
Rent	28	34.1%
Common	9	11.0%
Accommodation	3	3.7%
Marital status		
Married	45	54.9%
Single	18	22.0%
Common-law marriage	17	20.7%
Divorced	2	2.4%
Scholarship		
Basic	1	1.2%
High school	28	34.1%
Technician	41	50.0%
University	12	14.6%
BMI		
Normal	32	39.0%
Overweight	42	51.2%
Obese	7	8.5%

*BMI* body mass index

**Table 2:** Laboratory parameters evaluated in the general group of patients with SpA

	SpA patients *n* = 82	
	*n*	%
Calprotectin		
Negative	59	72.0
> 120 ng/ml	20	24.4
> 250 ng/ml	12	14.6
CRP		
< 0.5 mg/L	41	50
0.5–3.0 mg/L	70	85.4
3.0–9.0 mg/L	8	9.8
> 9.0 mg/L	3	3.7
ESR		
Normal	60	73.2
> 20 mm/h	21	25.6

*CRP* C-reactive protein, *ESR* erythrocyte sedimentation rate

Two or more gastrointestinal symptoms were found in 70.7% of patients. When applying the major screening criteria (Table 3), 48.7% of the individuals fulfilled at least one major criteria associated with a history of previous infection (*p* = 0.037). The 14.6% of patients presented rectal bleeding, 43.9% diarrhoea of more than 4 weeks, and no patients presented perianal disease. The association of rectal bleeding with active smoking (*p* = 0.021) was noteworthy. Likewise, rectal bleeding was associated with the diagnosis of AS (*p* = 0.050). On the other hand, the presence of diarrhoea was associated with abdominal pain (*p* = 0.018) and food intolerance (*p* = 0.045), specifically with the intake of grains and dairy (*p* = 0.028 and *p* = 0.01, respectively). It was also associated with high CRP levels and simultaneous abdominal pain (*p* = 0.041), as well as activity indices greater by ASDAS-ESR (*p* = 0.020).

**Table 3:** Description of SpA patients with gastrointestinal symptoms without diagnosis of IBD who fulfilled the IBD screening criteria

	SpA patients *n* = 82	
	*n*	**%**
Major criteria		
Rectal bleeding	12	14.6
Diarrhoea of more than 4 weeks	36	43.9
Perianal disease	0	0.0
Minor criteria		
Abdominal pain of more than 4 weeks	30	36.9
Iron deficiency anaemia	0	0.0
Iron deficiency	15	18.8
Extra intestinal manifestations		
Yes	2	2.5
Subtype of extra intestinal manifestation		
Aphthous stomatitis	2	2.5
Fever or low-grade fever	0	0.0
Unexplained weight loss	13	15.5
Family history of inflammatory bowel disease	0	0.0
Vitamin B12 deficiency	3	13.9

In addition, diarrhoea was associated with a history of smoking (*p* = 0.045) and previous gastrointestinal infection (*p* = 0.017). It was also associated with iron deficiency (*p* = 0.036) and abdominal distension (*p* = 0.030). Higher rates of diarrhoea were found in patients with biological treatment (*p* = 0.050). Finally, diarrhoea was associated with the simultaneous presence of elevated calprotectin (*p* = 0.001) and abdominal pain (*p* = 0.005).

Regarding minor criteria, they were found in 43.5%, associated with greater activity of the disease evaluated by the BASDAI scale (*p* = 0.043), BASFI index (*p* = 0.001), intake of meats (*p* = 0.041) and simultaneously elevated calprotectin levels (*p* < 0.050).

Within the minor criteria, abdominal pain was found in 36.9% of patients, being associated with simultaneous axial and peripheral involvement (*p* = 0.017), inflammatory lumbar pain (*p* = 0.010), enthesitis (*p* = 0.021), blood in the stool (*p* = 0.003), fatigue (*p* = 0.001) and abdominal distension (*p* = 0.001).

On the other hand, aphthous stomatitis and weight loss were found in 2.46% and 15.5% of patients, respectively. The weight loss associated with bleeding in the stool (*p* = 0.009) and faecal calprotectin higher than 250 ng/mL (*p* = 0.027). Patients with very high levels of calprotectin and moderate disease activity evaluated by ASDAS-CRP and ASDAS-ESR showed greater abdominal pain (*p* = 0.080 and *p* = 0.023, respectively). Finally, no cases of anaemia and fever were found. Vitamin B12 deficiency was found in 13.9% of individuals (Table 3).

**Fig. 1:**
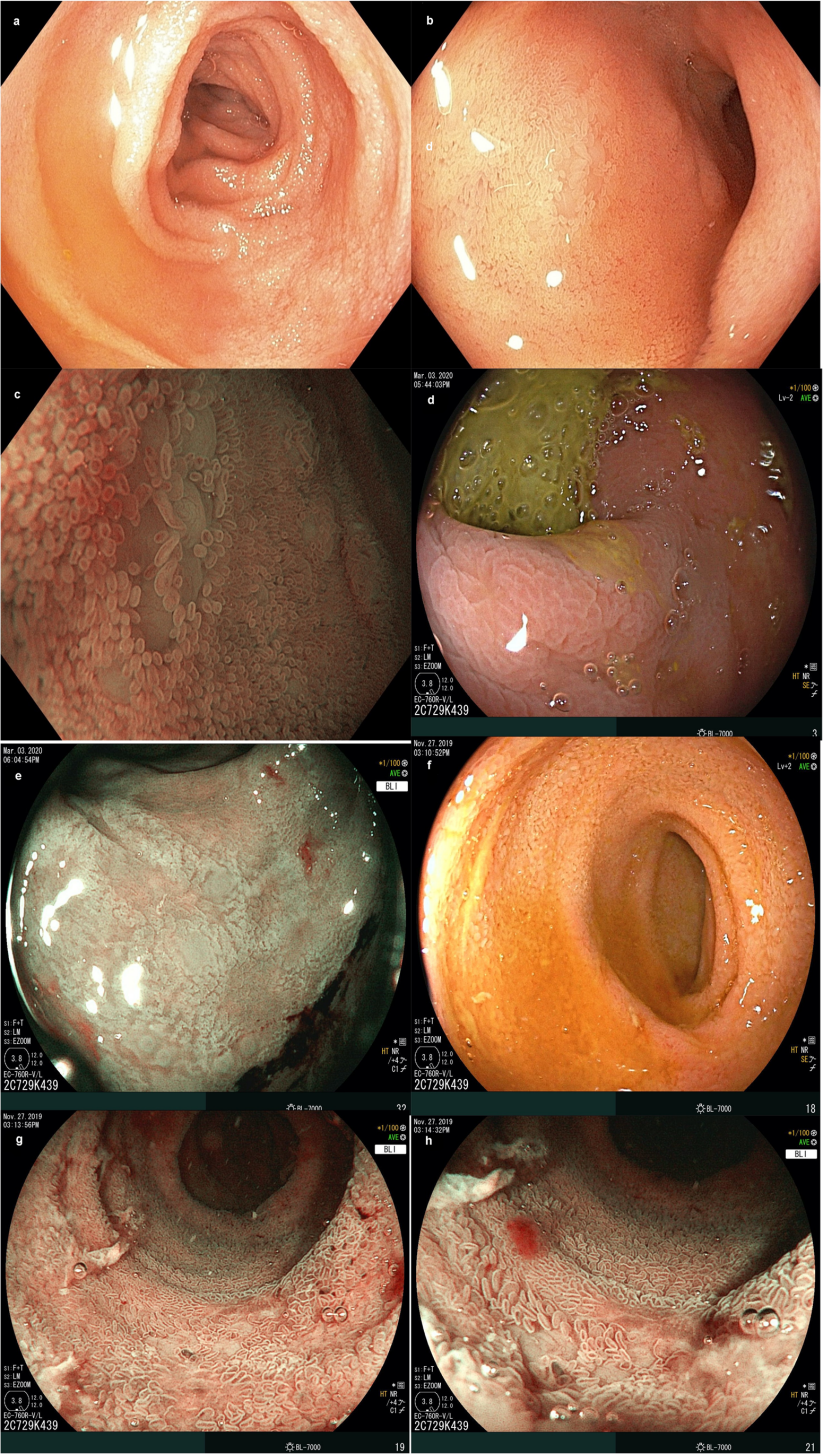
Ileocolonoscopy findings. **a** Distal ileum, visualisation with white light. **b** Magnification of the distal ileum. Areas of atrophy (white light). **c** Distal ileum, magnification with NBI digital chromoendoscopy. Areas of atrophy are seen with attenuation of the vascular pattern and loss of villi. **d** Sigmoid colon mucosa. White light, appreciating poorly defined irregular area. **e** Sigmoid colon mucosa. Digital chromoendoscopy BLI + magnification, appreciating area with loss of vascular pattern, superficial ulcers and thickening of the mucosa. **f** Distal ileum. White light with small erosions. **g** Distal ileum. BLI digital chromoendoscopy + magnification. Irregular ulcers with loss of vascular pattern and villus atrophy. Histological correlation with Crohn’s disease. **h** Distal ileum. BLI digital chromoendoscopy + magnification. Irregular ulcers with loss of vascular pattern and villus atrophy. Histological correlation with Crohn’s disease

**Fig. 2:**
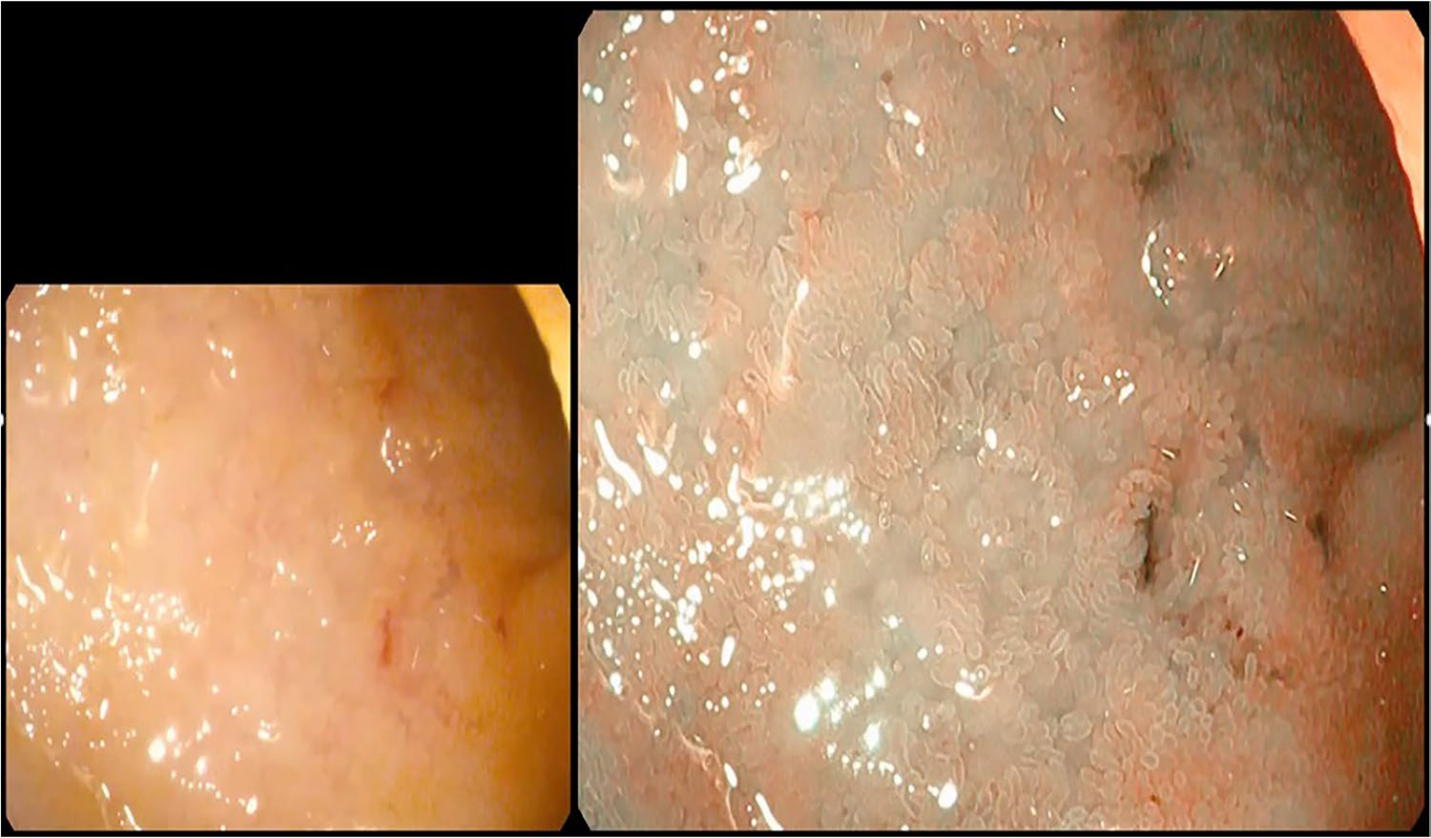
Comparison between white light (left) and digital chromoendoscopy (I-Scan 3) + magnification (right). Distal ileum with ulcers and denuded areas due to loss of villi

A group of patients, in addition to the screening criteria, was evaluated by ileocolonoscopy with digital chromoendoscopy with magnification and histological analysis to establish associations with the endoscopic and histological findings. In the evaluation of the sigmoid colon, mucosal changes were detected in 17.1%, loss of vascular pattern in 9.8%, erosions in 9.8%, ulcers in 2.4% and erythema in 2.1%. Regarding the ileum, mucosal changes were evidenced in 41.5%, villus atrophy in 36.6%, loss of vascular pattern in 29.3%, erosions in 9.8%, ulcers in 12.2% and erythema in 9.8% of the patients (Figs. 1 and 2).

At the ileum level, the histopathological analysis and diagnosis showed pattern and inflammation in the ileal mucosa in 41.5% and alteration in the architecture in 31.7% associated with the presence of rectal bleeding (*p* = 0.034; Supplementary Table 1).

The microscopic studies in the colon showed an inflammatory pattern in 31.7%, a chronic type in 26.8% and acute inflammation in 17.1% of the cases. Finally, rectal inflammation was reported in 19.5% and eosinophilia in 4.9% of the patients (Supplementary Table 1).

On the other hand, 70.7% of patients had at least one of the minor screening criteria, being significantly related to the inflammatory pattern of the colon (*p* = 0.029). The abdominal pain was present in 87.8% of the patients and associated with high rates of disease activity BASDAI (*p* = 0.023), ASDAS-CRP (*p* = 0.043) and inflammation in the ileum (*p* = 0.046). Finally, within this group of minor criteria, an alteration in ferritin was observed in 22% of the patients, being associated with the presence of chronic inflammation in the colon (*p* = 0.042).

In 17.1% of the cases, a decrease in the levels of vitamin B12 was detected, significantly associated with the presence of ulcers and acute inflammation in the ileum, *p* = 0.035 and *p* = 0.032, respectively. Similarly, at the level of the colon, the chronic inflammatory pattern was related to this same decrease (*p* = 0.048).

**Fig. 3:**
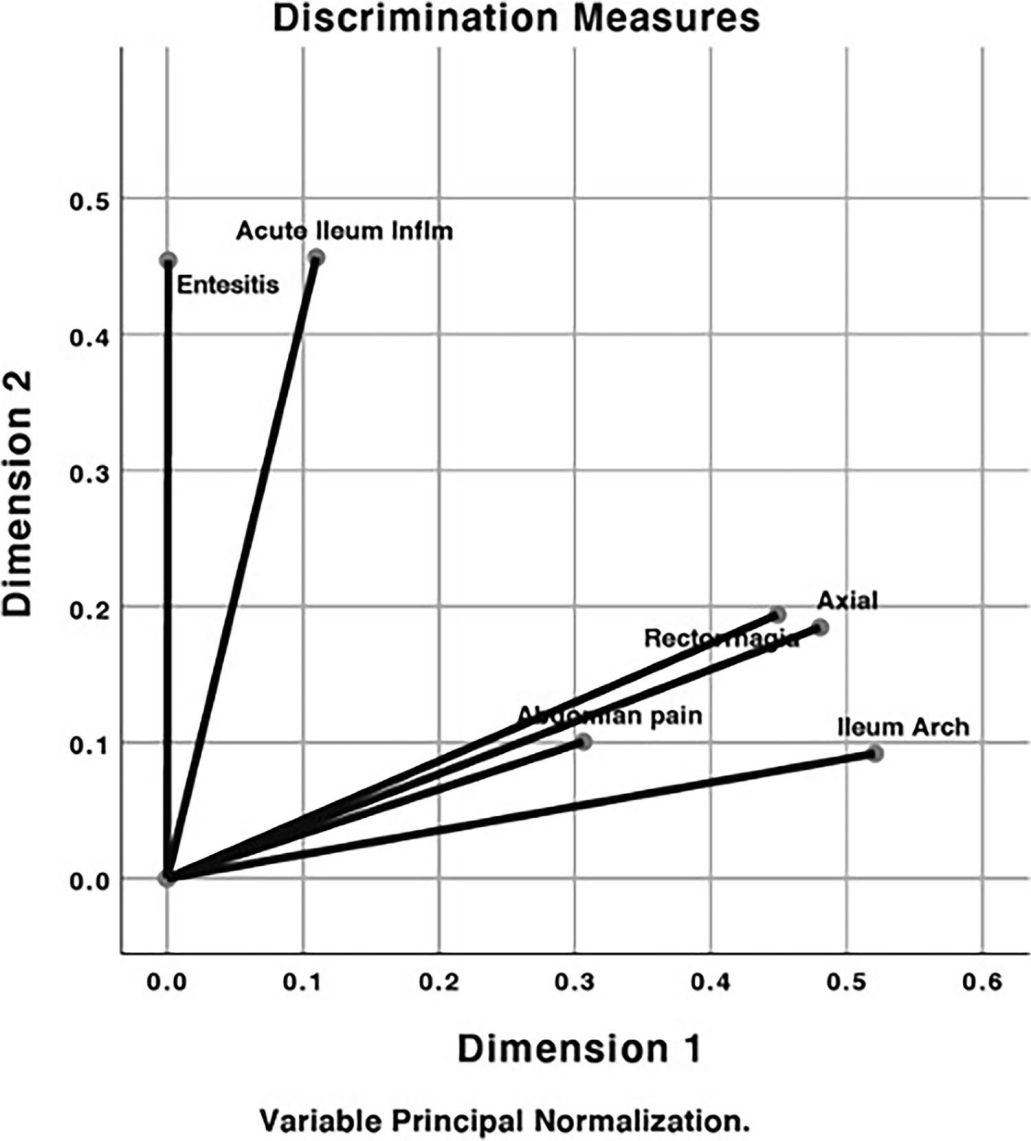
Multiple correspondence discriminant analysis. Relationship between the presence of rectal bleeding with axial involvement, enthesitis, acute inflammation of the ileum and a higher degree of disability in patients with SpA. Dim 1 grouped axial involvement (CC = 0.480), abdominal pain (CC = 0.306), rectal bleeding (CC = 0.449) and architecture alteration in the ileum (CC = 0.521), while the Dim 2 constituted by enthesitis (CC = 0.454) and acute inflammation of the ileum (CC = 0.457)

To perform, the multiple correspondence discriminant analyses were included axial involvement, enthesitis, acute inflammation of the ileum, abdominal pain, rectal bleeding and architecture alteration in the ileum. This model showed an internal consistency of 0.557 measured by Cronbach’s alpha. The model showed two dimensions. The model showed two dimensions: Dim 1 grouped axial involvement, abdominal pain, rectal bleeding and architecture alteration in the ileum, while the Dim 2 constituted by enthesitis and acute inflammation of the ileum (Fig. 3). The results are shown on a Cartesian plane, where the variables are represented as vectors which angles will be more acute the higher the level of correlation between them. Analysis allows for grouping variables with high correlation coefficients (CCs) and discriminating groups of patients with common characteristics. The length of each vector represents the CC of the variable within the group, which ranges from − 1.0 to + 1.0. A high contribution was considered when CC values were > 0.7, intermediate when 0.5–0.7 and low when < 0.5. All variables with CCs < 0.3 were excluded (Fig. 3 and Supplementary Fig. 1).

## Discussion

Several studies have estimated the occurrence of IBD in patients with SpA [10]. In our study, we found that about half of the individuals met, at least, one major criteria and was associated with a history of the previous infection, a characteristic finding in patients with SpA, as has been described since 1988 by Neumann V et al. [11].

The association observed between rectal bleeding and active cigarette consumption is striking, as has been described [12]. The association of cigarette smoking and Crohn’s disease proposes a potential interaction between harmful agents of the cigarettes that reach the intestine via an oral route as a risk mechanism [13].

Abdominal pain was found in more than a third of the patients, being associated with axial and peripheral involvement simultaneously, inflammatory lumbar pain, enthesitis and fatigue. This identifies a group of patients with a greater commitment to joint disease, information already corroborated by Silvio Danese et al., who described red flags suggestive of CD, included abdominal pain as one of the most representative symptoms [14].

In the group individuals with greater involvement and gastrointestinal symptoms, 31.7% of the patients met the major criteria of which the most important was of diarrhoea of more than 4 weeks associated with greater disease activity, a symptom previously reported in Colombian population [15].

Regarding rectal bleeding, those who were ruled out for haemorrhoidal disease did not reach 20%. However, the presence of rectal bleeding was associated with axial and peripheral involvement, which can guide us as a tool for early screening. Rectal bleeding was associated with high CRP levels, abdominal pain and higher activity indices, which has been supported in the literature [16, 17]. In these patients, abdominal pain was associated with higher indices of disease activity and mucosal alteration in the ileum. The concept that this is a warning symptom to be important in patients in whom inflammatory changes in the ileum are reported [18].

Oral lesions are frequent in patients with IBD, with a prevalence between 5 and 50% [19]. Aphthous stomatitis was found with frequencies lower than reported in IBD. It is important to consider the oral symptoms could precede intestinal manifestations in 60% of patients and, in many cases, lead to the diagnosis of the disease [20]. Furthermore, in active CD, they are associated with more oral signs [21].

The implementation of digital chromoendoscopy plus magnification was applied, finding changes in all intestinal segments, especially in the ileum, as the most important intestinal segment associated with SpA [22]. The most frequent were loss of vascular pattern and signs of acute and chronic inflammation, evidencing intestinal inflammatory involvement, similar to data reported from Leirisalo-Repo M, et al. as silent intestinal involvement [23].

In our study, we found two groups of patients: the first those with predominantly axial involvement who present with abdominal pain, rectal bleeding and architecture alteration in the ileum and the second patients with enthesitis and acute inflammation of the ileum; this association between higher enthesitis scores and IBD has been previously described [24]; however, this observation must be confirmed in subsequent studies.

In a relevant way, a trend was found between the presences of a chronic inflammatory pattern with diarrhoea, as has been shown in other studies [18]. Therefore, early endoscopic changes take on important relevance in association with gastrointestinal symptoms to generate a prompt opportunity for diagnosis, treatment and referral to gastroenterology [23].

The main limitation of our study is the cross-sectional design nature, given our results should be confirmed with an adequate follow-up focused on the evolution of intestinal involvement over time. Otherwise, we had a relatively small number of patients: due in part to the difficulty in recruiting patients in the early stages of the disease and considering delayed appointments and restrictions of the health system regarding timely referral to the specialist, as well as the large number of exclusion criteria that affect the recruiting phase. It could lead to the fact that we did not have a broad spectrum of gastrointestinal disease, which could affect the ability to discriminate the variables in our analysis.

To avoid selection bias during clinical evaluation, only expert rheumatologists performed it according to the ASAS classification criteria. One rheumatologist with the most clinical experience was defined as the gold standard. The study included measurements to avoid biases as a paired variable collection for gastrointestinal symptoms and medical history was done by asking specific questions by professionals, who were previously trained, with further validation by two gastroenterologists. At the same time, the trained personnel took the samples, transported and processed those using once-validated techniques and internal controls for each of them. A single person entered data for greater control. Nevertheless, additional studies with larger sample sizes and probabilistic statistical methods should confirm the present results.

## Conclusions

This is the first study of IBD screening criteria application in a group of Colombian patients with SpA without IBD, correlated with endoscopic and histologic findings. The main symptoms were diarrhoea of more than 4 weeks and abdominal pain, which were related to early manifestations of inflammatory bowel compromise and increased disease activity. The findings of subclinical inflammatory bowel involvement in ileum and colon were detected early, evaluated by colonoscopy plus digital chromoendoscopy and associated with some of these criteria. A good history and detailed physical examination can alarm clinicians about intestinal compromise in patients with SpA. This generates an opportunity to guarantee an adequate and early referral to the gastroenterology service from the rheumatology department, as well as potential therapeutic adjustments and nutritional habits and lifestyles.

## Supplementary Information

Below is the link to the electronic supplementary material.

Supplementary file1 (DOCX 286 KB)

## Data Availability

The data used to support the findings of this study are available from the corresponding author upon request.

## Strengths

This is the first study on these characteristics in a Colombian population with SpA, which generates important knowledge about our patients to make a diagnosis, select treatment and impact the population with SpA with inflammatory bowel involvement without a diagnosis of IBD early.
