# Supplementary material for: Implementation of screening criteria for inflammatory bowel disease in patients with spondyloarthritis and its association with disease and endoscopic activity
Source: Clin Rheumatol. 2022 Sep 2;42(2):415–22. doi: 10.1007/s10067-022-06297-7 (PMC9873707; doi:10.1007/s10067-022-06297-7)
Supplement: Supplementary file 1 — Supplementary file1 (DOCX 286 KB) [file 10067_2022_6297_MOESM1_ESM.docx]

**SUPPORTING INFORMATION**

**Materials and Methods**

**Methods:**

The medical record was retrieved to find information of gastrointestinal symptoms: diarrhea (defined as more than three depositions per day), stools with mucous, hematochezia, number of stools per day, abdominal pain, abdominal bloating, food intolerances, and weight loss. Patients with two or more of the mentioned gastrointestinal symptoms were referred to gastroenterology service. Of them, 58 were defined as having an indication for gastroenterological evaluation, and 41 underwent ileocolonoscopy with digital chromoendoscopy with magnification and histological analysis.

Pregnant and lactating women, and cancer patients, patients with other autoinflammatory diseases, autoimmune diseases, immunodeficiency, chronic pancreatitis, or chronic liver disease, and those who had received antibiotic treatment in the last 3 months were excluded from the study. Furthermore, those patients with SpA and concomitant IBD were excluded. All included patients were between 18 and 65 years old and signed informed consent for sample collection and ileocolonoscopy procedure. The patients were treated between December 2018 and December 2020.

**Ileocolonoscopy with digital chromoendoscopy with magnification** [1]**.**

Volume for colonoscopy was prepared with Travad Pik (Cali, Colombia; sodium picosulfate 10 mg + light magnesium oxide 3.5 g + citric acid 12 g), achieving adequate cleaning of all colonic and distal ileum tracts, measured with the Boston scale (9/9) [2].

**Ileocolonoscopy with digital chromoendoscopy with magnification** [1]**.**

Volume for colonoscopy was prepared with Travad Pik (Cali, Colombia; sodium picosulfate 10 mg + light magnesium oxide 3.5 g + citric acid 12 g), achieving adequate cleaning of all colonic and distal ileum tracts, measured with the Boston scale (9/9) [2].

Colonoscopies were performed under sedation with an anesthesiologist’s assistance by a gastroenterologist expert in diagnostic and therapeutic endoscopy [3] using Olympus EVIS EXERA III (CF- *HQ190L*/ I; Tokyo, Japan) or FUJI EC equipment - *760ZP*-V / L Zoom, ELUXEO 700 Series (Tokyo, Japan). This equipment allows performing electronic magnification of the mucosa and digital chromoendoscopy NBI (Olympus) and BLI (FUJI). Submucosal vascular pattern and stromal alterations were evaluated both in the villi of the ileum and in the pattern of the crypts of the colon. Multiple mucosa biopsies of the distal ileum and left colon were taken with a Jaw 4 Standard Capacity radial disposable forceps (Boston Scientific).

**Screening criteria** [4]**.**

**Major criteria**

- Rectal bleeding, unless hemorrhoidal semiology is evident and hemorrhoids are present on physical examination.
- Chronic diarrhoea (more than 4 weeks) of organic origin characteristics.
- Perianal disease: a set of anomalies that appear singly or in combination in the anus and rectum of patients with Crohn's disease.

**Minor criteria**

- Chronic abdominal pain (more than 4 weeks, persistent or recurrent).
- Iron deficiency anemia or iron deficiency.
- Extraintestinal manifestations (erythema nodosum, pyoderma gangrenosum, oral aphtha, or primary sclerosing cholangitis).
- Fever or low-grade fever without apparent focus and lasting more than a week.
- Unexplained weight loss.
- Family history of IBD.

**SpA activity evaluation.**

The disease clinimetry was calculated using Bath Ankylosing Spondylitis Disease Activity Index (BASDAI), Ankylosing Spondylitis Disease Activity Score (ASDAS) and Bath Ankylosing Spondylitis Functional Index (BASFI) [5, 6]. The evaluation of functional scores is validated for axSpA. For peripheral SpA (pSpA), currently, there are no validated indices that assess the activity and functionality of the disease.

**Faecal calprotectin measurement.**

Faecal calprotectin was measured through quantitative enzyme-linked immunosorbent assay according to the manufacturer's instructions: DiaSource-Quantitative Faecal Calprotectin KAPEPKT849®, cut-off > 120ng/mL, Louvain La Neuve, Belgium.

**High sensitivity CRP measurement.**

The test was performed by chemiluminescence (Immulite 1000, Siemens®) REF LKCRP1, according to the manufacturer's instructions; the reference values were 0-3 mg/dl (Munich, Germany).

**Determination of erythrocyte sedimentation rate (ESR).**

ESR was assessed with the Wintrobe method. Reference values used were greater than 20 mm/hour.

**Histological evaluation.**

The technique of paraffin embedding fine-cutting and routine staining with Hematoxylin-Eosin was used for distal ileum, sigmoid colon and rectum. An average of 18 to 24 levels per sheet were obtained from each sample. Special staining (Masson's trichrome, Gomory, PAS with and without diastase, Ziehl-Neelsen) or immunohistochemistry was performed as needed [7].

**Supplementary Table 1*.* Findings in the ileocolonoscopy in the gastrointestinal mucosa of patients with SpA**

|  | SpA patients  n = 82 | |
| --- | --- | --- |
|  | **n** | **%** |
| Rectus |  |  |
| Affected mucous | 7 | 17.1% |
| Erythema | 2 | 4.9% |
| Vascular pattern loss | 7 | 17.1% |
| Erosions | 4 | 9.8% |
| Ulcers | 1 | 2.4% |
| Inflammation | 6 | 14.6% |
| Sigmoid colon |  |  |
| Affected mucous | 7 | 17.1% |
| Erythema | 1 | 2.4% |
| Vascular pattern loss | 4 | 9.8% |
| Erosions | 4 | 9.8% |
| Ulcers | 1 | 2.4% |
| Ileum |  |  |
| Affected mucous | 17 | 45.9% |
| Erythema | 4 | 9.8% |
| Vascular pattern loss | 12 | 29.3% |
| Erosions | 4 | 9.8% |
| Ulcers | 5 | 12.2% |
| Atrophy of intestinal villous | 15 | 36.6% |
| Hemorrhoids | 9 | 22.0% |

**Supplementary Fig 1.**

1. **Multiple Correspondence Discriminant Analysis. Relationship between abdominal pain and the presence of higher disease activity (ASDAS-CRP and BASDAI) and inflammation in the ileum and alteration in vitamin B12 levels.**
2. **Discriminant analysis of multiple correlations. Relationship between Vit B12 hypovitaminosis and the presence of inflammation in the ileum, HLAB 27 and disease dysfunction.**


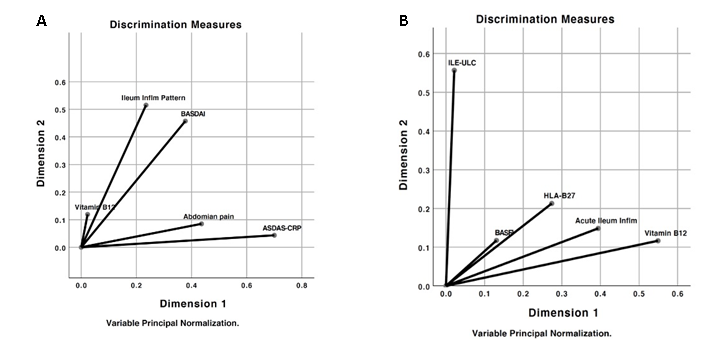


**Supplementary References**

1. Manfredi MA, Abu Dayyeh BK, Bhat YM, et al (2015) Electronic chromoendoscopy. Gastrointestinal Endoscopy 81:249–261. https://doi.org/10.1016/j.gie.2014.06.020

2. Calderwood AH, Jacobson BC (2010) Comprehensive validation of the Boston Bowel Preparation Scale. Gastrointestinal Endoscopy 72:686–692. https://doi.org/10.1016/j.gie.2010.06.068

3. Cotton P, editor. Sedation, analgesia and monitoring for endoscopy in ADEP and Safety [On line]: BP 2008. (2008) Sedation, Analgesia, and Monitoring for Endoscopy. In: Cotton PB (ed) Advanced Digestive Endoscopy: Practice and Safety. Blackwell Publishing, Ltd, Oxford, UK, pp 33–42

4. Sanz Sanz J, Juanola Roura X, Seoane-Mato D, et al (2018) Screening of Inflammatory Bowel Disease and Spondyloarthritis for Referring Patients Between Rheumatology and Gastroenterology. Reumatología Clínica (English Edition) 14:68–74. https://doi.org/10.1016/j.reumae.2017.07.001

5. Garrett S, Jenkinson T, Kennedy LG, et al (1994) A new approach to defining disease status in ankylosing spondylitis: the Bath Ankylosing Spondylitis Disease Activity Index. The Journal of rheumatology 21:2286–91

6. Lukas C, Landewé R, Sieper J, et al (2009) Development of an ASAS-endorsed disease activity score (ASDAS) in patients with ankylosing spondylitis. Annals of the rheumatic diseases 68:18–24. https://doi.org/10.1136/ard.2008.094870

7. Suvarna K, Layton C BJBT and P of HTE-BookPE 2018. (2019) Bancroft’s Theory and Practice of Histological Techniques. Elsevier
